# Continuation of Dabigatran Therapy in “Real-World” Practice in Hong Kong

**DOI:** 10.1371/journal.pone.0101245

**Published:** 2014-08-01

**Authors:** Mei Han Ho, Chi Wai Ho, Emmanuel Cheung, Pak Hei Chan, Jo Jo Hai, Koon Ho Chan, Esther W. Chan, Gilberto Ka Kit Leung, Hung Fat Tse, Chung Wah Siu

**Affiliations:** 1 Cardiology Division, Department of Medicine, Queen Mary Hospital, the University of Hong Kong, Hong Kong SAR, China; 2 Neurology Division, Department of Medicine, Li Ka Shing Faculty of Medicine, the University of Hong Kong, Hong Kong SAR, China; 3 Department of Pharmacology and Pharmacy, the University of Hong Kong, Hong Kong SAR, China; 4 Division of Neurosurgery, Department of Surgery, Li Ka Shing Faculty of Medicine, the University of Hong Kong, Hong Kong SAR, China; University of Bologna, Italy

## Abstract

**Background:**

Dabigatran, an oral direct thrombin inhibitor, possesses several advantages over warfarin that can in principle simplify the management of stroke prevention in atrial fibrillation (AF). Nonetheless it remains unclear whether these advantages can translate to clinical practice and encourage long-term therapy. The objective was to describe long-term dabigatran therapy for stroke prevention in AF and to identify risk factors for discontinuation of therapy.

**Methods and Results:**

We studied 467 consecutive Chinese patients (72±11 years, male: 53.8%) with a mean CHA_2_DS_2_-VASc score of 3.6 prescribed dabigatran for stroke prevention in AF from March 2010 to September 2013. Over a mean follow-up of 16 months, 101 patients (21.6%) permanently discontinued dabigatran. The mean time-to-discontinuation was 8 months. The most common reason for discontinuation was dyspepsia (30.7%), followed by other adverse events (17.8%) such as minor bleeding (8.9%), major gastrointestinal bleeding (7.9%), and intracranial hemorrhage (1%). Other reasons included dosing frequency (5.9%), fear of side effects (4.0%), lack of laboratory monitoring (1.0%), and cost (1.0%). Multivariable analysis revealed that low baseline estimated glomerular filtration rate (*p* = 0.02), absence of hypertension (*p = *0.01), and prior use of a proton-pump inhibitor (*p* = 0.02) and H_2_-receptor blocker (*p* = 0.01) were independent predictors of drug discontinuation. In addition, there were altogether 9 ischemic strokes (1.5%/years), 3 intracranial hemorrhages (0.5%/year), and 24 major gastrointestinal bleedings (4.1%/year).

**Conclusion:**

Dabigatran discontinuation is very common amongst Chinese AF patients. This reveals a management gap in the prevention of stroke in AF.

## Introduction

Atrial fibrillation (AF) is the most common sustained cardiac arrhythmia encountered in clinical practice.[1], [2] Patients with AF have a 5-fold higher risk of ischemic stroke.[1] While the role of long-term oral anticoagulation therapy in the form of vitamin K antagonist for stroke prevention in AF has been established for many years, such therapy is grossly underutilized amongst Chinese: only 15% of Chinese AF patients eligible for long-term anticoagulation therapy actually receive it.[3] This may be related to a higher risk of intracranial hemorrhage amongst Chinese prescribed a vitamin K antagonist,[4]–[6] a widely held belief of low ischemic stroke risk amongst Chinese AF patients,[7] ^,^ [8], [9] and the potential for herb-drug interaction due to the high prevalence of herbal consumption in Chinese.[10]

Dabigatran, an oral direct thrombin inhibitor, was approved for stroke prevention in patients with non-valvular AF by the US Food and Drug Administration in 2010, and European Medicines Agency in 2011. The drug has been commercially available in Hong Kong since 2010. Dabigatran possesses several long-awaited advantages over warfarin, such as a fixed dosing regimen that requires no anticoagulation monitoring or dose adjustment, and few drug-food and drug-drug interactions. This can, in principle, simplify the management of stroke prevention in AF. Nonetheless it remains unclear whether these advantages can enhance continuation of therapy in clinical practice, and thus realize the projected clinical benefits. In the RE-LY study, the 1-year and 2-year discontinuation rate of dabigatran was ∼15% and ∼20% higher than the corresponding rates for warfarin of only ∼10% and ∼16%.[11] In this study, the objective was to describe continued dabigatran therapy for stroke prevention in AF, and identify the reasons and risk factors for discontinuation of therapy in a cohort of Chinese AF patients.

## Methods

### Patients

Between March 2010 and September 2013, 500 patients at Queen Mary Hospital prescribed Dabigatran were identified through the computer-based clinical management system. Patients in whom dabigatran was prescribed for pulmonary embolism, venous thrombosis or post-operative prophylaxis were excluded. The final analysis included 467 Chinese patients with AF.

### Study Design and Definitions

This was a single-center, registry-based, observational study. The study protocol was approved by the Institutional Review Board of Queen Mary Hospital. Informed consents were not obtained from the patients given the registry nature of the study; however, all patient records/information was anonymized and de-identified prior to analysis. Data pertaining to baseline demographics, cardiovascular risk factors, gastrointestinal conditions and medications were extracted from the electronic records of the Clinical Management System Database. Hypertension was defined as resting systolic and/or diastolic blood pressure ≥140/90 mmHg on two occasions or prescription of anti-hypertensive agents. Diabetes mellitus was defined as a serum fasting glucose ≥7.0 mmol/l or prescription of anti-diabetic medication. Congestive heart failure was defined according to the Framingham Heart Study, and left ventricular (LV) systolic dysfunction was defined as LV ejection fraction (EF) ≤40%. Obesity was defined as a body mass index (BMI) ≥30 kg/m^2^. Glomerular filtration rate was estimated (eGFR) using the following formula: eGFR  = 175× (Scr/88.4)^-1.154^× Age^-0.203^×0.742 (if female) ×1.212 (if African American).[12] Ischemic stroke was defined as a neurological deficit of sudden onset that persisted for >24 hours, corresponding to a vascular territory in the absence of primary hemorrhage that could not be explained by other causes (trauma, infection, vasculitis). Stroke was confirmed by computerized axial tomography or magnetic resonance imaging of the brain.[13], [14] Intracranial hemorrhage was diagnosed in the presence of new onset neurological symptoms with radiological confirmation (computerized axial tomography scan or magnetic resonance imaging).[15] The CHADS_2_ score (Congestive heart failure, Hypertension, Age≥75 years, Diabetes, previous Stroke), more recently, the CHA_2_DS_2_-VASc score (CHA_2_DS_2_-Vascular disease, Age 65-74 years, Sex category), and HAS-BLED score were calculated.

### Study Outcomes

The primary endpoint was permanent discontinuation of dabigatran during the follow-up period. Events such as temporary discontinuation due to bleeding or need for surgery and death within the follow-up period were not considered as discontinuation. Other endpoints included the occurrence of clinical events such as ischemic events, intracranial hemorrhage, and bleeding. Major bleeding was defined as fatal bleeding, any bleed that caused a reduction in haemoglobin level ≥2 g/dL, requirement for transfusion of at least 2 units of whole blood or red cells, and/or symptomatic bleeding in a critical area or organ. Minor bleeding included any bleeding not considered as major bleeding.

### Statistical Analysis

Continuous variables were expressed as mean ± SD whereas categorical variables were reported as frequencies and percentages. Statistical comparisons of the baseline clinical characteristics were performed using Chi-square test, Student's t test, Mann-Whitney U test or Fisher's exact test as appropriate. Crude incidence event rates were calculated as percentage per person-years. The Cox proportional hazards regression model was used to calculate the hazard ratios (HRs) of some predictive factors and their 95% confidence intervals (CIs) for dabigatran discontinuation. Variables with a *p*-value <0.10 as determined in the univariate analyses were added into the multivariable regression model. A *p*-value <0.05 was considered statistically significant. Data analysis was performed using IBM SPSS Statistics 21 (SPSS, Inc., Chicago, Illinois).

## Results

### Baseline Characteristics

Table 1 summarizes the baseline clinical characteristics of the study cohort.

**Table 1:** Baseline characteristics.

	All (n = 467)	Dabigatran discontinuation		*P* value
		Yes (n = 101)	No (n = 366)	
Age, y	72±11	71±13	72±10	0.86
Male	248 (53.1)	53 (52.5)	195 (53.3)	0.89
Mean CHADS_2_	2.08±1.39	1.95±1.46	2.12±1.37	0.28
Mean CHA_2_DS_2_-Vasc	3.58±1.86	3.44±1.93	3.62±1.84	0.37
Mean HAS-BLED	2.02±1.07	1.95±1.16	2.04±1.05	0.68
Ever-smoker	89 (19.1)	21 (20.8)	68 (18.6)	0.62
Medical Conditions				
CHF/LV dysfunction	111 (23.8)	33 (32.7)	78 (21.3)	0.02*
Hypertension	312 (66.8)	58 (57.4)	254 (69.4)	0.02*
Diabetes mellitus	124 (26.6)	25 (24.8)	99 (27.0)	0.64
Prior stroke/TIA	135 (28.9)	24 (23.8)	111 (30.3)	0.20
Intracranial hemorrhage	4 (0.9)	0 (0.0)	4 (1.1)	0.58
Myocardial infarction	26 (5.6)	8 (7.9)	18 (4.9)	0.24
Bleeding history	53 (11.3)	15 (14.9)	38 (10.4)	0.21
GERD	9 (1.9)	2 (2.0)	7 (1.9)	1.00
Esophagitis	15 (3.2)	3 (3.0)	12 (3.3)	1.00
Gastritis	50 (10.7)	11 (10.9)	39 (10.7)	0.95
Peptic ulcer disease	30 (6.4)	4 (4.0)	26 (7.1)	0.25
Drug induced dyspepsia	4 (0.9)	0 (0.0)	4 (1.1)	0.58
Functional dyspepsia	6 (1.3)	3 (3.0)	3 (0.8)	0.12
Prior *H. pylori* infection	28 (6.0)	8 (7.9)	20 (5.5)	0.36
Baseline Creatinine, µmol/L	93±31	103±43	90±26	0.02*
Baseline eGFR, mL/min/1.73m^2^	66.6±20.0	61.9±19.9	67.9±19.8	0.01*
Prior Medications				
Warfarin	196 (42.0)	43 (42.6)	153 (41.8)	0.89
Aspirin	311 (66.6)	63 (62.4)	248 (67.8)	0.31
Thienopyridine	101 (21.6)	19 (18.8)	82 (22.4)	0.44
NSAIDs	152 (32.5)	32 (31.7)	120 (32.8)	0.83
Proton pump inhibitor	149 (31.9)	42 (41.6)	107 (29.2)	0.02*
Antacids	215 (46.0)	53 (52.5)	162 (44.3)	0.14
H2-receptor blocker	281 (60.2)	69 (68.3)	212 (57.9)	0.06
Concurrent Medications				
Aspirin	70 (15.0)	19 (18.8)	51 (13.9)	0.22
Thienopyridine	16 (3.4)	2 (2.0)	14 (3.8)	0.54
NSAIDs	34 (7.3)	5 (5.0)	29 (7.9)	0.31
Proton pump inhibitor	196 (42.0)	46 (45.5)	150 (41.0)	0.41
H2-receptor blocker	194 (41.5)	41 (40.6)	153 (41.8)	0.83
Amiodarone	41 (8.8)	14 (13.9)	27 (7.4)	0.04*

*p<0.05. Abbreviations: CHF: congestive heart failure; eGFR, estimated glomerular filtration rate; GERD, gastroesophageal reflux disease; NSAIDs, non-steroidal anti-inflammatory drugs; TIA: transient ischemic attack.

A total of 467 patients were included in the final analysis. The mean age was 72±11 years with a male predominance (53.1%). The mean CHADS_2_ score was 2.1±1.4, the mean CHA_2_DS_2_-Vasc score was 3.6±1.9, and the mean HAS-BLED score was 2.0±1.1. Of the 467 patients, 312 patients had hypertension (66.8%), 135 had prior stroke or transient ischemic attack (28.9%), and 124 had diabetes mellitus (26.6%). The prevalence of pre-existent upper gastrointestinal disease was varied: drug-induced dyspepsia (0.9%), functional dyspepsia (1.3%), gastroesophageal reflux disease (1.9%), esophagitis (3.2%), *Helicobacter pylori* infection (6.0%), peptic ulcer disease (6.4), and gastritis (10.7%). While the defaulted dose of dabigatran was 150 mg two times daily, majority of patients (391 patients, 83.7%) in the present cohort were prescribed 110 mg two times daily as in other Asian countries. Only 47 patients (10.1%) were prescribed the standard regime (150 mg two times daily). In addition 26 patients (5.6%) were prescribed 75 mg two times daily mostly because of renal impairment and elderly age.

### Drug Discontinuation

**Figure 1:**
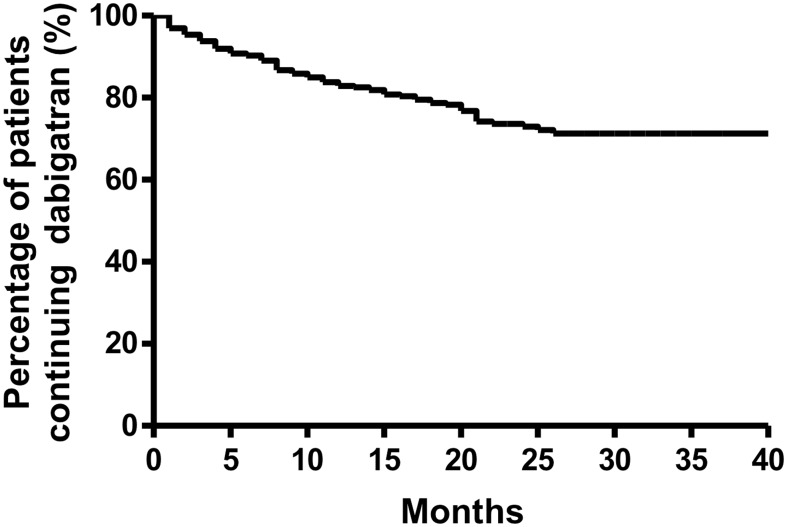
Kaplan Meier analysis of drug discontinuation rate.

**Figure 2:**
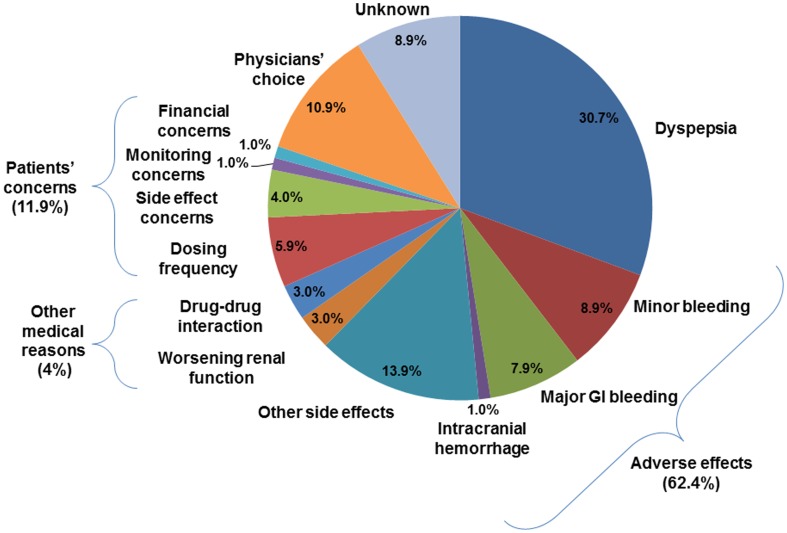
Reasons of discontinuation of dabigatran.

Over a mean follow-up period of 16±10 months (a total of 583.5 dabigatran-patient-years), dabigatran was discontinued in 101 of 467 patients (21.6%). The mean and median time-to-discontinuation were 7.5±6.7 months and 5.5 months (interquartile range: 2–12 months) respectively (Figure 1). Adverse events and side effects accounted for most instances of discontinuation (62.4%)(Figure 1). Dyspepsia was the most common reason for dabigatran discontinuation (30.7%), followed by bleeding complications: major gastrointestinal bleeding in 8 patients (7.9%), and intracranial hemorrhage in one (1.0%). Other side effects that resulted in dabigatran discontinuation included skin rash, pruritus, reflux symptoms, headache and generalized discomfort. In addition, potential drug-drug interaction (3.0%) and worsening of renal function (3.0%) were medical reasons for dabigatran discontinuation. Patient concerns such as dosing frequency (5.9%), fear of side effects (4.0%), monitoring concerns (1.0%) and financial concerns (1.0%) accounted for 11.9% of dabigatran discontinuation (Figure 2).

Table 1 summarizes the baseline clinical characteristics of patients who discontinued therapy (21.6%) and those who continued therapy (78.4%). There were no statistically significant differences in age, gender, proportion of those prescribed warfarin, CHADS_2_, CHA_2_DS_2_-VASc or HAS-BLED scores between those who discontinued and those who continued dabigatran therapy. Nonetheless those who discontinued treatment had a higher proportion of congestive heart failure/LV systolic dysfunction (32.7% vs. 21.3%, *p* = 0.018), poorer baseline renal function (eGFR: 62±20 mL/min/1.73 m^2^ *vs.* 68±20 mL/min/1.73 m^2^, *p* = 0.008; and serum creatinine: 103±43 µmol/L vs. 90±26 µmol/L, *p* = 0.021), but a lower proportion of hypertension (57.4% vs. 69.4%, *p* = 0.024). Interestingly, although there was no significant difference in the incidence of previously documented upper gastrointestinal conditions, those who discontinued dabigatran were more likely to have been previously prescribed a proton-pump inhibitor (41.6% vs. 29.2%, *p* = 0.018). Likewise, in patients who discontinued dabigatran a higher proportion had previously taken amiodarone (13.9%, vs. 7.4%, *p* = 0.041). In multivariable analysis, only prior use of proton pump inhibitors, and H_2_-receptor blockers, and baseline eGFR were independent predictors of dabigatran discontinuation, while prior stroke or transient ischemic attack and hypertension were independently associated with continued of therapy (HR: 0.62, 95% CI: 0.39–0.98, *p* = 0.042)(Table 2).

**Table 2:** Associations between baseline factors and dabigatran discontinuation in Chinese AF patients.

	Univariate Analysis		Multivariable Analysis	
	HR (95% CI)	*p-*value	HR (95% CI)	*p-v*alue
Age	0.99 (0.98–1.01)	0.35		
Male	1.01 (0.68–1.49)	0.97		
CHADS_2_	0.90 (0.78–1.04)	0.16		
CHA_2_DS_2_-Vasc	0.94 (0.84–1.04)	0.22		
HAS-BLED	0.92 (0.77–1.11)	0.40		
Ever-smoker	1.09 (0.67–1.76)	0.72		
Medical conditions				
CHF/LV dysfunction	1.58 (1.04–2.40)	0.03*	1.37 (0.87–2.14)	0.17
Hypertension	0.62 (0.42–0.93)	0.02*	0.54 (0.35–0.83)	0.01*
Diabetes mellitus	0.89 (0.57–1.40)	0.62		
Prior stroke/TIA	0.65 (0.41–1.04)	0.07	0.62 (0.39–0.98)	0.04*
Bleeding history	1.43 (0.83–2.48)	0.20		
Gastritis	1.06 (0.57–1.99)	0.85		
Baseline eGFR	0.99 (0.98–0.99)	0.02*	0.99 (0.98–0.99)	0.02*
Prior Medications				
Warfarin	0.92 (0.62–1.36)	0.67		
Aspirin	0.86 (0.57–1.28)	0.46		
Thienopyridine	0.84 (0.51–1.39)	0.51		
NSAIDs	0.98 (0.65–1.50)	0.94		
Proton pump inhibitor	1.65 (1.11–2.46)	0.01*	1.64 (1.09–2.46)	0.02*
Antacid	1.31 (0.89–1.93)	0.18		
H2-receptor blocker	1.55 (1.02–2.36)	0.04*	1.80 (1.16–2.82)	0.01*
Concurrent Medications				
Aspirin	1.37 (0.83–2.26)	0.22		
Proton pump inhibitor	1.10 (0.74–1.62)	0.65		
H2-receptor blocker	0.89 (0.60–1.33)	0.57		
Amiodarone	1.73 (0.99–3.05)	0.06	1.41 (0.79–2.55)	0.25

*p<0.05. Abbreviations: CHF, congestive heart failure; eGFR, estimated glomerular filtration rate; LV, left ventricular; NSAIDs, non-steroidal anti-inflammatory drugs; TIA, transient ischemic attack.

### Clinical Events

During follow-up, there were 14 cerebral ischemic events (9 ischemic strokes or 5 transient ischemic attacks) with an annual incidence of 2.4% (Table 3). The annual risk of ischemic stroke and transient ischemic attack was 1.5% and 0.9% respectively. In addition, there were altogether 98 bleeding complications with an annual incidence of 16.8%. Amongst these, 31 constituted major bleeding events (5.3%/year) including 24 major gastrointestinal bleeds (4.1%/year), and 3 intracranial hemorrhages (0.5%/year). In the cases of major gastrointestinal bleeding, 6 patients (25%) were taking concomitant antiplatelet agents, 4 were on non-steroidal anti-inflammatory agent, 2 were on amiodarone. In addition, 12 of the 24 patients (50%) had a history of a pre-existent upper gastrointestinal condition. Amongst the 3 patients with intracranial hemorrhage, none was prescribed concomitant antiplatelet therapy, but 1 was taking a non-steroidal anti-inflammatory agent. There were 34 instances of minor gastrointestinal bleeding (5.8%/year), and 33 of minor bleeding at another site such as the urinary tract, respiratory tract and subcutaneous tissue (5.7%/year). There were 10 deaths (1.7%/year) during the follow-up period: 3 were due to a cardiovascular cause (0.5%/year). Furthermore, warfarin use prior to the initiation of dabigatran was not associated with any clinical events.

**Table 3:** Clinical events.

		Annual event rate (%)
**Ischemic events**		
Ischemic stroke/TIA, n (%)	14 (3.0)	2.4
Ischemic stroke, n (%)	9 (1.9)	1.5
TIA, n (%)	5 (1.1)	0.9
Myocardial Infarction, n (%)	3 (0.6)	0.5
Unstable angina, n (%)	1 (0.2)	0.2
**Bleeding events**		
Any bleeding, n (%)	98 (21.0)	16.8
Major bleeding, n (%)	31 (6.6)	5.3
Intracranial, n (%)	3 (0.6)	0.5
Gastrointestinal, n (%)	24 (5.1)	4.1
Other, n (%)	4 (0.9)	0.7
Minor bleeding, n (%)		
Gastrointestinal, n (%)	34 (7.3)	5.8
Other, n (%)	33 (7.1)	5.7
**Gastrointestinal symptoms**		
Dyspepsia	77 (16.5)	13.2
Reflux	32 (6.9)	5.5
**Death**, n (%)		
Cardiovascular, n (%)	3 (0.6)	0.5
All-cause, n (%)	10 (2.1)	1.7

## Discussion

Dabigatran is the first oral anticoagulant approved by the Food and Drug Administration for stroke prevention in non-valvular AF, more than 50 years after warfarin was first approved. The drug has overcome many of the major shortcomings of warfarin, and more importantly has been proven to be comparable to warfarin at a dose of 110 mg two times daily and superior to warfarin at a dose of 150 mg two times daily in preventing stroke in patients with non-valvular AF.[11] The translation of these research findings to clinical benefits requires continuation of therapy. In the RE-LY study, the 2-year discontinuation rate of dabigatran was >20%, much higher than for warfarin (16.6%). In the present study, 21.6% of Chinese AF patients, more than 1 in 5 patients, prescribed dabigatran for stroke prevention discontinued the drug over a mean follow-up of 16 months, a much higher rate than that of the RE-LY study.[11] Nonetheless, the finding was similar to real-world studies in Japan and Hong Kong which reported discontinuation rates of 21.7% in 300 patients after a median follow-up of 263 days and 25.4% in 122 patients after a median follow-up of 310 days respectively.[16], [17]

In our cohort, a vast majority of dabigatran discontinuation was related to adverse events and/or side effects. Amongst these, dyspepsia was the single most common reason, accounting for up to 30% of instances (6.6% of patients, compared with 2% in the RE-LY study).[11] Similarly, Miyamoto and colleagues observed that discontinuation of dabigatran was mainly due to dyspepsia, followed by deteriorating renal function, then minor bleeding.[16] On the other hand, a multi-center study reported that high cost accounted for half of the discontinuation whereas gastrointestinal side effects such as dyspepsia and flatulence came next.[18] Of note, dabigatran-induced dyspepsia was much more common in the present cohort than in the RE-LY study (16.5% vs. 11.8% in patients prescribed dabigatran 110 mg two times daily, and 11.3% in those prescribed 150 mg two times daily).[11] In the Long-term Multicenter Extension of Dabigatran Treatment in Patients with Atrial Fibrillation (RELY-ABLE) study, which included almost 50% of patients originally randomized to dabigatran in the RE-LY trial and who continued to receive the same blinded dabigatran dose,[19] 21.5% of those prescribed 110 mg two times daily and 20.7% of those prescribed 150 mg two times daily reported dyspepsia.[20]

Interestingly, while previously diagnosed upper gastrointestinal conditions did not predict dabigatran discontinuation, prior use of proton pump inhibitors and H_2_-receptor blockers did. This suggests that some patients might have had pre-existent upper gastrointestinal pathology that required treatment with a proton pump inhibitor and H_2_-receptor blocker, and that was subsequently exacerbated by dabigatran. Nonetheless it has been recently reported that simple measures such as taking dabigatran with a meal, and use of a proton pump inhibitor, H_2_-receptor blockers and/or non-prescription antacids can significantly alleviate symptoms of dyspepsia.[20] In contrast, history of hypertension and stroke was associated with lower discontinuation risk. This may be explained by physicians' perception of higher stroke risk among this group of patients. Other adverse clinical events, particularly bleeding complications, also contributed to dabigatran discontinuation: the risk of bleeding in the present cohort appeared to be higher than in the RE-LY trial: 16.8% per year for major and minor bleeding compared with 14.6%/year in dabigatran 110 mg two times daily, and 16.4%/year in 150 mg two times daily. More importantly, the rate of intracranial hemorrhage was 0.5%/year in the present cohort, substantially higher than in the RE-LY trial (0.2% to 0.3%/year).[11] In the recently published sub-study of RE-LY that included an Asian-only population,[21] the annual risk of intracranial hemorrhage was only 0.23%/year in patients on dabigatran 110 mg two times daily and 0.45%/year in patients on dabigatran 150 mg twice daily. Even more alarmingly, the rate of major gastrointestinal bleeding was 4.11%/year in our cohort, substantially higher than that of both the Asian (0.96%/year to 1.15%/year) and non-Asian population (1.14%/year to 1.69%/year) in the RE-LY study.[21] While some studies in the real world setting documented very few bleeding events, ranging from 3.3% to 5.2%,[16], [18], [22] Michel and colleagues revealed that 29% of patients receiving dabigatran had experienced bleeding events, predominantly minor ones.[23] In particular, Ho and colleagues reported the total bleeding and intracranial hemorrhagic risks of 22.95% and 1.64%, respectively, which were even higher than that in our cohort.[17] This high incidence of bleeding complications may be related to a high prevalence of pre-existent upper gastrointestinal disorders and the concomitant use of anti-platelet agents and/or non-steroidal anti-inflammatory drug.

The results of our study have several important clinical implications.

First, Chinese AF patients prescribed dabigatran had a high drug discontinuation rate due to upper gastrointestinal side effects or bleeding complications. This may be related to the higher prevalence of pre-existent, undiagnosed upper gastrointestinal disorders that were then subsequently revealed or exacerbated by dabigatran. Thus, screening for upper gastrointestinal symptoms prior to prescription of dabigatran may allow early identification of upper gastrointestinal pathology, and thereby prevent bleeding complications and improve treatment compliance. Second, as dyspepsia and bleeding complications can be managed by proper adjustment of medication and some simple measures, additional support and counseling for patients on dabigatran therapy by healthcare professionals may likewise improve compliance with therapy.

### Limitations

This observational study had some limitations. First, our study was limited by the small sample due to a single-hospital registry, although to our knowledge, this is the largest published Chinese AF cohort prescribed Dabigatran.

Second, the decision to discontinue dabigatran was made by the individual patient and their attending physician. Third, this was a hospital-based study using single-hospital registry data and did not include patients with a milder form of ischemic stroke, and/or bleeding complications who did not require hospitalization.

### Conclusion

Amongst Chinese patients prescribed dabigatran for stroke prevention in AF, the discontinuation rate is substantial, mainly due to adverse events and side effects. This study provides data on the main reasons for dabigatran discontinuation in Chinese AF patients. This may allow implementation of measures such as patient education to improve drug compliance and thus ensure continuation of therapy.
